# Gene Flow, Subspecies Composition, and Dengue Virus-2 Susceptibility among *Aedes aegypti* Collections in Senegal

**DOI:** 10.1371/journal.pntd.0000408

**Published:** 2009-04-14

**Authors:** Massamba Sylla, Christopher Bosio, Ludmel Urdaneta-Marquez, Mady Ndiaye, William C. Black

**Affiliations:** 1 Department of Microbiology, Immunology and Pathology, Colorado State University, Fort Collins, Colorado, United States of America; 2 Departement de Biologie Animale, Faculte des Sciences et Techniques, Universite Cheikh Anta DIOP de Dakar, Dakar, Senegal; University of California Berkeley, United States of America

## Abstract

**Background:**

*Aedes aegypti*, the “yellow fever mosquito”, is the primary vector to humans of the four serotypes of dengue viruses (DENV1-4) and yellow fever virus (YFV) and is a known vector of Chikungunya virus. There are two recognized subspecies of *Ae. aegypti sensu latu* (*s.l.*): the presumed ancestral form, *Ae. aegypti formosus (Aaf)*, a primarily sylvan mosquito in sub-Saharan Africa, and *Ae. aegypti aegypti (Aaa)*, found globally in tropical and subtropical regions typically in association with humans. The designation of *Ae. aegypti s.l.* subspecies arose from observations made in East Africa in the late 1950s that the frequency of pale “forms” of *Ae. aegypti* was higher in populations in and around human dwellings than in those of the nearby bush. But few studies have been made of *Ae. aegypti s.l.* in West Africa. To address this deficiency we have been studying the population genetics, subspecies composition and vector competence for DENV-2 of *Ae. aegypti s.l.* in Senegal.

**Methods and Findings:**

A population genetic analysis of gene flow was conducted among 1,040 *Aedes aegypti s.l.* from 19 collections distributed across the five phytogeographic regions of Senegal. Adults lacking pale scales on their first abdominal tergite were classified as *Aedes aegypti formosus (Aaf)* following the original description of the subspecies and the remainder were classified as *Aedes aegypti aegypti (Aaa)*. There was a clear northwest–southeast cline in the abundance of *Aaa* and *Aaf*. Collections from the northern Sahelian region contained only *Aaa* while southern Forest gallery collections contained only *Aaf*. The two subspecies occurred in sympatry in four collections north of the Gambia in the central Savannah region and *Aaa* was a minor component of two collections from the Forest gallery area. Mosquitoes from 11 collections were orally challenged with DENV-2 virus. In agreement with the early literature, *Aaf* had significantly lower vector competence than *Aaa*. Among pure *Aaa* collections, the disseminated infection rate (DIR) was 73.9% with a midgut infection barrier (MIB) rate of 6.8%, and a midgut escape barrier (MEB) rate of 19.3%, while among pure *Aaf* collections, DIR = 34.2%, MIB rate = 7.4%, and MEB rate = 58.4%. Allele and genotype frequencies were analyzed at 11 nuclear single nucleotide polymorphism (SNP) loci using allele specific PCR and melting curve analysis. In agreement with a published isozyme gene flow study in Senegal, only a small and statistically insignificant percentage of the variance in allele frequencies was associated with subspecies.

**Conclusions:**

These results add to our understanding of the global phylogeny of *Aedes aegypti s.l.*, suggesting that West African *Aaa* and *Aaf* are monophyletic and that *Aaa* evolved in West Africa from an *Aaf* ancestor.

## Author Summary

We conducted a population genetic study with 1,040 *Aedes aegypti sensu latu* (*s.l.*) collected from 19 sites distributed across the five phytogeographic regions of Senegal. Adult mosquitoes without pale scales on their first abdominal tergite were classified as *Aedes aegypti formosus* (*Aaf*) and those having pale scales as *Aedes aegypti aegypti* (*Aaa*). We found the two forms distributed along a northwest–southeast cline. Northern Sahelian collections contained only *Aaa* while the southern Forest gallery collections consisted of only *Aaf*. The two subspecies were sympatric in four collections north of The Gambia. *Aaa* was a minor component of two collections from the Forest gallery area. Eleven of these collections were fed a dengue-2 virus–infected bloodmeal. Consistent with the early literature, *Aaf* had lower vector competence than *Aaa*. In agreement with a recently published isozyme gene flow study in Senegal, analyzes of allele frequencies indicated only a small, nonsignificant percentage of the variance associated with subspecies. These results improve our understanding of the global phylogeny of *Aedes aegypti s.l.*, suggesting that West African *Aaa* and *Aaf* are monophyletic and that *Aaf*, the black “sylvan” species, is the ancestor of *Aaa*, the lighter “domestic” species in West Africa.

## Introduction

*Aedes aegypti*, the “yellow fever mosquito”, is the primary vector to humans of the four serotypes of dengue viruses (DENV1-4), yellow fever virus (YFV) and is a known vector of Chikungunya virus. Dengue is a major public health problem in tropical regions of the world, causing millions of dengue fever and hundreds of thousands of dengue hemorrhagic fever cases annually [1]. In endemic areas the annual number of cases has risen steeply since the 1950s [2]. With multiple serotypes circulating in endemic areas, 100 million infections of dengue fever (DF) occur annually, including up to 500,000 cases of the more severe form of disease called dengue hemorrhagic fever (DHF) with a case fatality rate of up to 5% [3]. Despite the development of a safe, effective YFV vaccine, yellow fever remains an important health risk in sub-Saharan Africa and tropical South America [4],[5]. The WHO estimates there are 200,000 cases and 30,000 deaths attributable to YFV infection each year, most of which occur in Africa [6].

There are two recognized subspecies of *Ae. aegypti s.l.*: the presumed ancestral form, *Ae. aegypti formosus (Aaf)*, a primarily sylvan mosquito in sub-Saharan Africa, and *Ae. aegypti aegypti (Aaa)*, found globally in tropical and subtropical regions typically in association with humans. The designation of *Ae. aegypti s.l.* subspecies arose from observations made in East Africa in the late 1950s that the frequency of pale “forms” of *Ae. aegypti* was higher in populations in and around human dwellings than in those of the nearby bush [7]. The implied correlation between color and behavior prompted Mattingly [8] to revisit the biology and taxonomy of *Ae. aegypti*. He described *formosus* (Walker) as a subspecies of *Ae. aegypti* that was restricted to sub-Saharan Africa and in West Africa “is the only form known to occur except in coastal districts and in one or two areas of limited island penetration.” He also suggested that it most frequently breeds in natural containers such as tree holes and feeds on wild animals. Mattingly also stated that, in addition to the dark-scaled parts of the body being generally blacker, “*ssp. formosus* never has any pale scales on the first abdominal tergite.” The type form of *Ae. aegypti aegypti* was alternatively defined as “either distinctly paler and browner (at least in the female) than *ssp. formosus* or with pale scaling on the first abdominal tergite or both.” He also suggested that *Aaa* breeds in artificial containers provided by humans, will breed indoors, and has a preference for feeding on human blood [9]. McClelland [10] made a comprehensive study of differences in scale patterns in the abdominal dorsum in 74 *Ae. aegypti s.l.* collected from 69 different worldwide locations. He concluded that many of Mattingly's subspecies distinctions were not always clear cut in Africa, the only region in the world where both forms are found. In East Africa, pure *Aaa* or *Aaf* collections as defined by both color and behavior were found but there were also collections where the subspecies were mixed. In areas of sympatry, he found intermediate forms, with peridomestic habits and a wide range of pale scaling. Populations widely overlapped in the extent of pale scaling. McClelland [10] concluded that, with a large enough sample size, populations could be distinguished on the basis of body color, although peridomestic populations overlapped with the distributions of both *Aaa* and *Aaf* populations. Body color alone, however, was unreliable as a means to assign individuals to a particular subspecies and instead, he recommended using the number of pale scales on the first abdominal tergite.

Later, mark-release-recapture studies in Kenya [11] demonstrated that immature mosquitoes collected from sylvan, peridomestic, or domestic breeding containers showed an overwhelming preference for their respective habitat as adults. In contrast, in West Africa, mosquitoes morphologically consistent with *Aaf* were found breeding domestically indoors in Nigeria [12] and Gabon [13]. Therefore, the classic behavioral/habitat descriptions given by Mattingly [8] for these two subspecies were not valid throughout Africa. In eastern Kenya, genetic crosses between *Aaf* and *Aaa* showed that preferences for endophily had a strong genetic component [14]. These authors speculated that these sympatric populations remained behaviorally and morphologically distinct because of adaptations that limited genetic exchange. *Aaf* rarely entered houses, and the authors proposed that those that did would not be likely to oviposit in water jars but would instead seek natural breeding sites in the forest. They speculated that the offspring of those that oviposit in water jars would not be adapted to surviving in the low nutritional content of drinking water. Conversely, they argued that gravid *Aaa* rarely enter the forest, and were not therefore attracted to tree holes. If they oviposited there, the larvae would not be adapted to avoiding predators found in natural containers. Those larvae that survived to adults would be anthropophilic and unlikely to find a suitable host. It was further hypothesized that the subspecies evolved allopatrically, and that *Aaa* was reintroduced into East Africa after adaptation to human habitats. Therefore these layers of behavioral differences were fully developed when the subspecies came into contact again, greatly restricting gene flow between them. Laboratory experiments crossing *Aaa* and *Aaf* from Kenya showed no evidence of assortative mating [15]. Furthermore, there was no decrease in fecundity in hybrids, nor any morphological defects.

**Figure 1:**
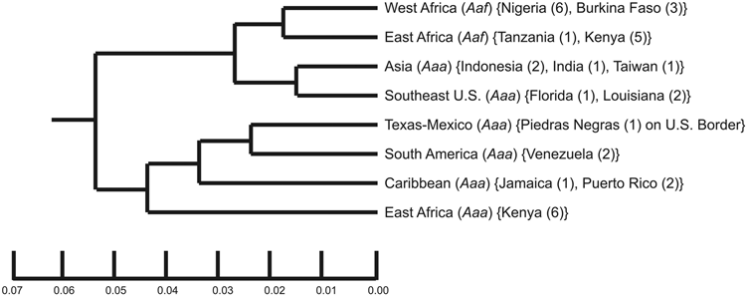
Genetic relationships among 34 worldwide collections of *Ae. aegypti s.l.* Each clade is labeled according to the original names followed by the country or location where the material was collected and, in parentheses, the number of collections. Modified from [25].

The monumental works of Tabachnick, Powell, Munstermann and Wallis [16]–[27] on the global population genetics and vector competence of *Ae. aegypti s.l.* showed that collections made throughout the species distribution fell into one of two clades (Figure 1). One clade contained *Aaa* from East Africa, South America, the Caribbean and Texas/Northeastern Mexico suggesting that these New World populations were derived from East Africa. The other clade contained Asian and Southeastern U.S. *Aaa* and a basal clade consisting of *Aaf* from East and West Africa. This tree topology suggested therefore independent New World and Asian introductions. Their parallel work with Beaty [17]–[19] on vector competence suggested that West African *Aaf* had lower competence for YFV than other global collections of *Aaf* and *Aaa*.

Despite the importance of these early groundbreaking studies they had, in retrospect, a number of deficiencies. They did not use the number of pale scales on the first thoracic tergite [9] to identify individual mosquitoes. Instead, whole *Ae. aegypti s.l.* collections were classified as either *Aaa* or *Aaf* based upon geographic origin, collection location (indoor *Aaa* vs. outdoor *Aaf*) and/or their general body coloration of “light” (*Aaa*) or “dark” (*Aaf*). Furthermore, they assumed that all West African *Ae. aegypti* were *Aaf*. Thus notice in Figure 1 that no *Aaa* were sampled from West Africa. This assumption was based upon Mattingly's [8] claim that in West Africa “*formosus* is the only form known to occur except in coastal districts and in one or two areas of limited island penetration.” But this statement was based largely upon collections from Ghana and Burkina Faso. Finally, all early vector competence work was based upon the Asibi strain of YFV. No work was done with DENV because dengue was not a prevalent disease at that time. In order to address these deficiencies, we have been studying the population genetics, subspecies composition and vector competence for DENV-2 of *Ae. aegypti s.l.* in Senegal. Here we report an analysis of 1,040 *Aedes aegypti sensu latu* (*s.l.*) from 19 collections distributed across the 5 phytogeographic regions of Senegal.

## Materials and Methods

### *Aedes aegypti* collections and extraction of DNA

**Figure 2:**
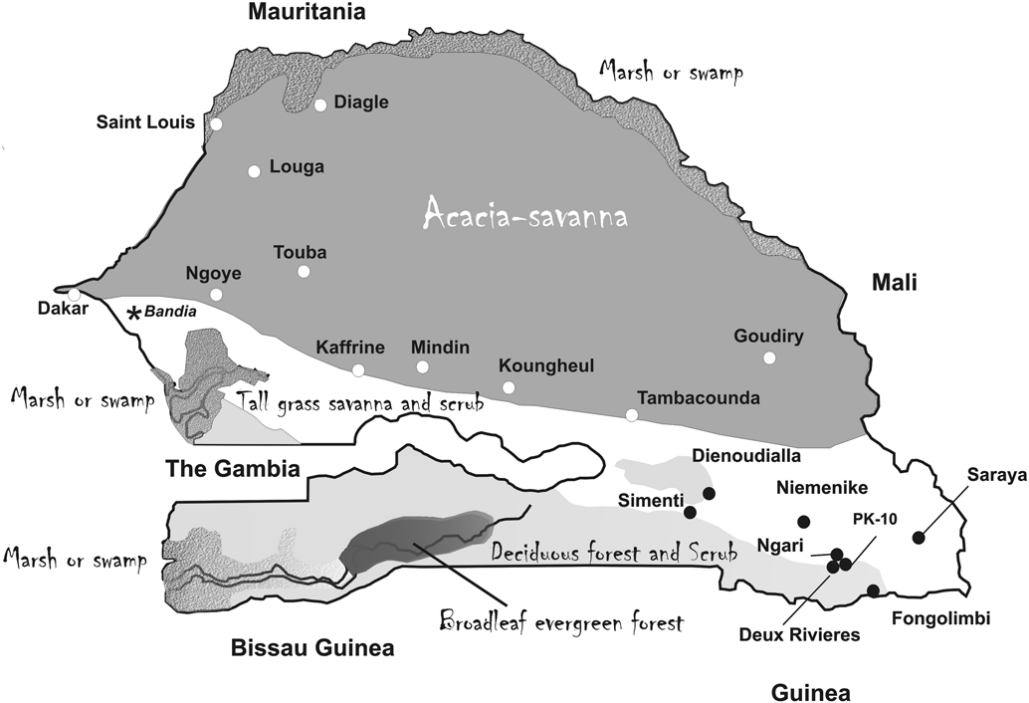
*Aedes aegypti s.l.* collection sites and associated sample sites in Senegal. Predominant vegetation zones are also shown.

From January 8, 2005–July 20, 2007 we collected *Ae. aegypti s.l.* immature stages (larvae and pupae) and eggs from the 19 locations in Senegal listed in Table 1 and mapped in Figure 2. At each urban and rural site, we collected immature stages from at least 30 different breeding sites in each of three different, distant locations at least 100 m apart. Breeding sites consisted of water storage containers and discarded trash such as plastic pails, tires, and cans. In the forest gallery sites of PK10 and Deux Rivieres, immature stages were collected from treeholes and from the discarded husks of *Saba senegalensis* (Apocynacea) which collect water during the rainy season. Eggs collection were also made using ten ovitraps in both of these forest gallery sites.

**Table 1:** Name, date, phytogeographic region, location, habitat and sample sizes of collection sites in Senegal.

City	Date collected	Phytogeographic region	Latitude (N)	Longitude (W)	Habitat	N^a^	N(SNP)^b^	N(VC)^c^
Saint-Louis	7/1/2007	Sahel	16° 1′32.44″	16°30′17.94″	Urban	26	26	83
Dígale	7/2/2007	Sahel	16°10′60.00″	15°45′0.00″	Rural Village	58	63	18
Louga	7/1/2007	Sahelo-sudan	15°36′55.03″	16°13′17.56″	Urban	58	56	-
Dakar	1/8/2005	Sahelo-sudan	14°44′59.97″	17°27′59.12″	Urban	61	46	54
N'goye	6/29/2007	Sudano-sahelian	14°36′51.25″	16°24′42.79″	Rural Village	59	58	52
Touba	4/16/2007	Sudano-sahelian	14°51′33.67″	15°52′43.80″	Urban	73	67	-
Mindin	7/16/2006	Sudanian	14° 3′58.55″	15°17′58.76″	Rural Village	36	36	-
Kaffrine	7/16/2006	Sudano-sahelian	14° 6′23.83″	15°33′7.25″	Urban	43	37	-
Koungheul	7/16/2006	Sudano-sahelian	13°58′33.49″	14°48′15.11″	Urban	52	48	-
Tambacounda	7/16/2006	Sudanian	13°46′23.13″	13°40′38.35″	Urban	105	58	50
Saraya	7/18/2006	Sudanian	12°49′60.00″	11°45′0.00″	Urban	25	54	-
Dienoudialla	7/17/2006	Sudanian	13°12′52.05″	13°6′43.15″	Rural Village	26	57	-
Goudiry	7/8/2007	Sudano-sahelian	14°11′13.02″	12°42′43.91″	Urban	58	60	58
Niemenike	7/17/2006	Sudanian	13°0′25.52″	12°32′48.14″	Rural Village	69	59	49
Ngari	11/20/2006	Sudanian	12°38′0.57″	12°14′59.77″	Rural Village	57	49	51
PK10	11/20/2006	Sudanian	12°36′0.09″	12°14′0.25″	Forest Gallery	40	59	35
Deux rivières	11/20/2006	Sudanian	12°38′0.20″	12°14′0.15″	Forest Gallery	83	51	38
Simenti	7/20/2007	Sudanian	13° 1′59.72″	13°17′58.77″	Rural Village	58	58	-
Fongolimbi	7/23/2006	Sudano-Guinean	12°24′44.88″	12°0′41.76″	Rural Village	53	56	26
TOTAL						1040	998	514

a N = number of mosquitoes examined for number of white scales on the first abdominal tergite.

b N(SNP) = number of F_1_ mosquitoes in the SNP genotype assays.

c N(VC) = number of F_1_ mosquitoes in the vector competence assays.

Eggs and immature stages were returned to the laboratory where they were reared to adults and then identified to species [28]. *Aedes aegypti s.l* were further identified as *Aaa* or *Aaf* based upon the number of pale scales on the first abdominal tergite [10]. If the first abdominal tergite lacked pale scales (McClelland's F range [10]) it was scored as *Aaf* and was otherwise scored as *Aaa*. These adults were provided access to sugar, allowed to mate for three days; males were then aspirated, and stored at −80°C. Every third day, over a two-week period, sugar was removed from the cages 24 h prior to bloodfeeding on mice. Bloodfed females were then given constant access to wet paper towels as an oviposition substrate. After two weeks females were aspirated and stored at −80°C. DNA was obtained from individual adults by salt extraction [29], suspended in 300 µl of TE buffer (10 mM Tris-HCl, 1 mM EDTA pH 8.0), and stored at −80°C.

### Vector competence

Mosquito collections were characterized for vector competence using an immunofluorescence assay (IFA) at 14 days post-oral challenge. The DENV-2 strain used was dengue 2 JAM1409 which was isolated in 1983 in Jamaica [30] and belongs to the American Asian genotype [31]. This DENV-2 strain was used rather than one from West Africa because we wished to compare vector competence data in *Ae. aegypti* from Senegal with all of our other collections including our standard susceptible Dengue 2 Susceptible on 3 chromosomes (*D2S3*) strain and our resistant Dengue 2 Midgut Escape Barrier (*D2MEB*) strains [32]; all of which have been characterized with JAM1409. All procedures for growing virus in 14 day cell culture, quantifying the virus, and infecting mosquitoes with membrane feeders covered with sterile hog-gut are published [33]. *D2S3* [32] served as a positive control to test for consistency in the quality and quantity of DENV-2 preparation and infection. Undiluted virus titers ranged from 7.5–8.5 log_10_ infectious virus/mL.

After exposure to the infectious bloodmeal, fully engorged mosquitoes were removed from the feeding carton and held for 14 days at a constant 27°C and 80% relative humidity in an insectary with a 12-hour photoperiod. Heads and abdomens were assayed for infection by IFA using a mouse derived primary monoclonal antibody directed against a flavivirus E gene epitope [34],[35]. Heads were checked first for DENV-2 infections. If the head was uninfected, the abdomen was checked for infection.

### SNP discovery

**Figure 3:**
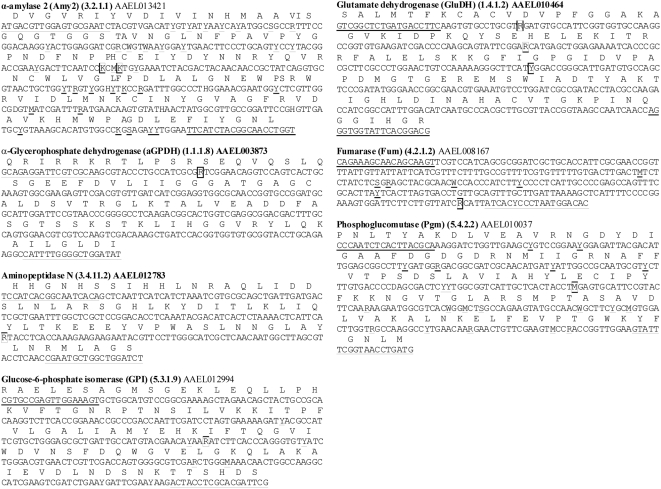
The amplified region of each of the 7 nuclear genes. PCR primer sites are underlined, all SNP sites are underlined, and the selected SNP is placed in a box.

Table 2 lists the primers and annealing temperatures for the eight gene regions from which we identified SNPs. Figure 3 shows the locations of SNPs in the amplified regions. These gene regions were amplified in the 57 *Ae. aegypti* listed in Table 3. Amplified products were screened for polymorphisms with Single Stranded Conformation Polymorphism (SSCP) analysis [29]. All novel SSCP genotypes were then sequenced to screen for SNPs. These sequences were then assembled into a single dataset and translated to assess whether each SNP encoded a synonymous or replacement substitution. Once a SNP locus was selected it was assigned the name of the gene followed by a numeric label indicating its distance in nucleotides from the adenine in the ATG start site.

**Table 2:** Sequences of primers used for PCR amplification of the eight gene regions in *Ae. aegypti s.l.* from Senegal.

Gene Name (E.C. No.)	Vector Base #	Forward Primer	Reverse primer	Amplicon size (bp)
α-Amylase (3.2.1.1)	AAEL013421	ATGACGTTGGAGTGCGAATC	ACCAGGTTGCCGTAGATGAA	350
a-Glycerophosphate dehydrogenase (1.1.1.8)	AAEL003873	GCAGAGGATTCGTCGCAA	ATATCCAGCCCCAAAATG	258
Aminopeptidase N (3.4.11.2)	AAEL012783	TCCATCACGGCAATCACA	AGATCCAGCCAGCATTCG	203
Fumarase (4.2.1.2)	AAEL008167	CAGAAAGCAACAGCAAGT	GTGTCCATTAGGGAGTGAT	282
Glucose-6-phosphate Isomerase (5.3.1.9)	AAEL012994	CGTGCCGAGTTGGAAAGT	CGAATCGTGCGAGGTAGT	239
Glutamate dehydrogenase (1.4.1.2)	AAEL010464	GTCGGCTCTGATGACCTTC	CGTCCGTAAATACCACCCT	312
Phosphoglucomutase (5.4.2.2)	AAEL010037	CCCAATCTCACTTACGCA	CATCAGGTTACCGAAATAC	593
Trypsin (early) (3.4.21.4)	AAEL007818	CCCAAAGCCAACAACCT	TTTYGTCCAACTCCAGCA	510–523

**Table 3:** Geographic origin, sex, and sample sizes of *Aedes aegypti s.l.* used to screen for SNPs.

Collection Location	Females	Males
*Ae. aegypti formosus* Deux Rivieres	4	9
*Ae. aegypti formosus* Ngari	0	7
*Ae. aegypti formosus* Pk10 strain	8	7
*Ae. aegypti aegypti* Dakar	15	7
Total	27	30

### SNP genotype identification

Genotypes at SNP loci were detected using allele specific PCR. Genotypes were determined in a single-tube PCR using two different “allele-specific” primers, each of which contained a 3′ nucleotide corresponding to one of the two alleles and an opposite primer that amplified both alleles. Allele specific primers were manufactured (Operon Inc., Huntsville, AL) with 5′ tails [36],[37] designed to allow discrimination between SNP alleles based on size or melting temperature. Primer sequences are provided in Table 4. An intentional transversion mismatch was introduced three bases in from the 3′ end of allele specific primers to improve specificity and each allele specific primer differed by a transition at this site [38]. Melting curve PCR was performed as previously described [39].

**Table 4:** Oligonucleotides used for allele specific PCR.

Gene Name	SNP locus	Oligonucleotide sequences (5′ end)	Oligonucleotide sequences (3′ end)
α-Amylase	Amy2.447Gf	**5′-GCGGGCAGGGCGGCGGGGGCGGGGCC**	ACCGAACGACTTCAATGC**G**-3′
	Amy2.447Tf	**5′-GCGGGC**	ACCGAACGACTTCAATAC**T**-3′
	Amy2.447r	5′-CCAGCAGTTACGCACCTGATAG-3′	
	Amy2.450f	5′-AACTTCCCTGCAGTCCCC-3′	
	Amy2.450Tr	5′-[long tail]	TAGTCGTAGATTTCAGA**A**-3′
	Amy2.450Gr	5′-[short tail]	TAGTCGTAGATTTCAAA**C**-3′
a-Glycerophosphate	αGPDH.55f	5′-GCAGAGGATTCGTCGCAA-3′	
dehydrogenase	αGPDH.55Gr	5′-[long tail]	GTGACTGGACCTGTTCCTA**C**-3′
	αGPDH.55Ar	5′-[short tail]	GTGACTGGACCTGTTCCCA**T**-3′
Aminopeptidase N	Apn.1938Gf	5′-[long tail]	TCACTCTAAAACTCATTGA**G**-3′
	Apn.1938Af	5′-[short tail]	TCACTCTAAAACTCATTAA**A**-3′
	Apn.1938r	5′-GAGCGATGCCCAAGGAAC-3′	
Fumarate hydratase	Fum.-294Gf	5′-[long tail]	GGAAAGTGGATTCTTCTTGTTAGC**G**-3′
	Fum.-294Af	5′-[short tail]	GGAAAGTGGATTCTTCTTGTTAAC**A**-3′
	Fum.-294r		
Glucose-6-phosphate	Gpi.1,500Gf	5′-[long tail]	GCTGATTGCCATGTACGAACACCA**G**-3′
Isomerase	Gpi.1,500Af	5′-[short tail]	GCTGATTGCCATGTACGAACACTA**A**-3′
	Gpi.1,500r	5′-CGTCCCAGATGACACCCT-3′	
Glutamate	GlutDH.507Gf	5′-[long tail]	GATGACCTTCAAGTGTGCCTGCTT**G**-3′
Dehydrogenase	GlutDH.507Af	5′-[short tail]	GATGACCTTCAAGTGTGCCTGCCT**A**-3′
	GlutDH.507r	5′-ATGYTCCGAATACTGCTTGGG-3′	
	GlutDH.567Gf	5′-[long tail]	CCCCAAGCAGTATTCGCA**G**-3′
	GlutDH.567Af	5′-[short tail]	CCCCAAGCAGTATTCGTA**A**-3′
	GlutDH.567r	5′-CGGTCCRATGAAGCCCTTTT-3′	
	GlutDH.627Cf	5′-[long tail]	TGTCCAAAAAGGGCTTCCT**C**-3′
	GlutDH.627Tf	5′-[short tail]	TGTCCAAAAAGGGCTTCTT**T**-3′
	GlutDH.627r	5′-CCCATATCGGGAGCKGGCA-3′	
Phosphoglucomutase	Pgm.954Cf	5′-[long tail]	GTCATTGCTCACTACGT**C**-3′
	Pgm.954Af	5′-[short tail]	GTCATTGCTCACTACGT**A**-3′
	Pgm.954r	5′-CTGTTGGCATACTTCTGGC-3′	
Trypsin (early)	TrypEarlIf	5′-[long tail]	GGCTACCGCATAACCCTGAACCACA-3′
	TrypEarlDf	5′-[short tail]	CTACCGCATAACCATGAACC-3′
	TrypEarlr	5′-TGGCTGAGTCCCAGAAGG-3′	

The sequences of the short and long tails are provided in bold for the first gene only. The 3′ allele specific nucleotide is bold and the mismatch at the third nucleotide from the 3′ end is underlined.

### Statistical analysis of haplotype and allele frequencies

Variation in allele frequencies among and within years, subspecies, phytogeographic regions, vegetation zones and habitats was determined by analysis of molecular variance (AMOVA) using the computer program Arlequin 3.01 [40]. This program also estimated pairwise F_ST_ values and Slatkin's linearized F_ST_ [F_ST_/(1−F_ST_)] [41] among collections and computed the significance of the variance components associated with each level of genetic structure by a nonparametric permutation test with 100,000 pseudoreplicates [40]. Pairwise linearized F_ST_ values were used to construct a dendrogram among all collections by means of unweighted pair-group method with arithmetic averaging analysis [42] in the NEIGHBOR procedure in PHYLIP3.5C [43]. Wright's F-Statistics were calculated using Weir and Cockerham's method [44].

## Results

### Subspecies distribution

**Figure 4:**
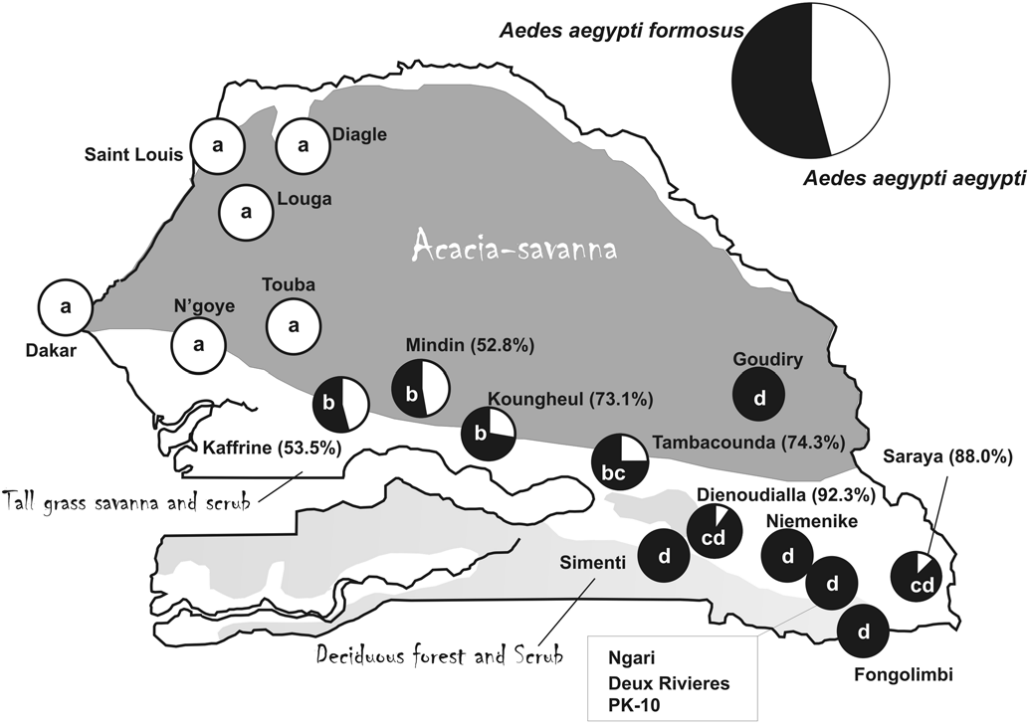
Distribution of *Aaa* or *Aaf* in Senegal. Pairwise Fisher's Exact Tests were performed on all collections. Strains with equivalent rates have the same labels and these were significantly different from one another.

Figure 4 shows the proportion and distribution of mosquitoes classified as *Aaa* or *Aaf* in the 19 collection sites. This figure suggests a northwest-southeast cline in the abundance of the two subspecies. Six collections from the Sahelian region in northwest Senegal where the primary vegetation type is *Acacia*-Savannah contained only *Aaa*. Six collections from the southern Forest gallery area in southern Senegal where the primary vegetation type is deciduous forest and scrub consisted of only *Aaf* (Ngari, PK-10 and Deux Rivieres are placed under a single pie chart in Figure 4). Only *Aaf* was found in Goudiry in the central *Acacia*-Savannah region. The two subspecies were sympatric in four sites north of The Gambia in the central Savannah region containing predominantly tall grass savanna and scrub and in Dienoudialla and Saraya in the southern Forest gallery area. Letters in the pie charts in Figure 4 indicate the results of pairwise 2×2 heterogeneity χ^2^ tests. Four statistically homogeneous groups were detected. Group ‘a’ are the pure *Aaa* collections while group ‘d’ are the pure *Aaf*, and the Dienoudialla and Saraya collections, groups ‘b’ and ‘c’ overlap and contain all of the collections in which the two subspecies are sympatric.

### Vector competence

**Figure 5:**
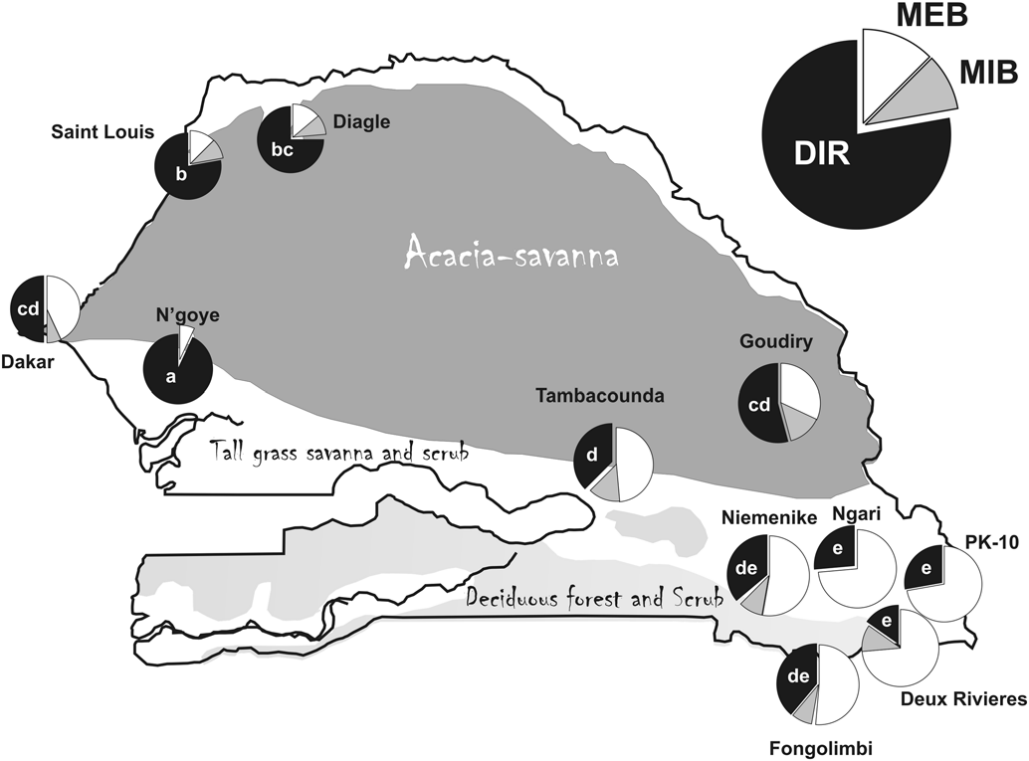
Vector competence of *Ae. aegypti s.l.* collections in Senegal. Disseminated infection rate (DIR) appears in black, midgut infection barrier rate (MIB) appears in grey, and midgut escape barrier rate (MEB) appears in white. Pairwise Fisher's Exact Tests were performed on all collections. Strains with equivalent rates have the same labels and these were significantly different from one another. Sample sizes = 50–65 females.

We incorporated our standard *D2S3* strain [32] as a positive control and standard refractory *D2MEB* [32] strain as a negative control. The Disseminated Infection Rate (DIR) was 92.3% in *D2S3* and 51.2% in *D2MEB* (sample sizes = 65 and 80 females, respectively). Figure 5 shows the proportion and distribution of mosquitoes with a disseminated infection (DIR), a midgut infection barrier (MIB) and a midgut escape barrier (MEB). There is a northwest-southeast cline in the susceptibility of *Ae. aegypti s.l.* populations. Northwestern *Aaa* collections have a high disseminated infection rate (DIR) while southeast *Aaf* collections have a low DIR associated with a MEB. Letters in the pie charts in Figure 5 indicate the results of pairwise 2×2 heterogeneity χ^2^ tests. Five statistically homogeneous groups were detected. N'goye (group ‘a’) had a higher DIR than the other 10 collections. Group ‘b’ contains the pure *Aaa* collections from the Sahel. Group ‘e’ contains the pure *Aaf* collections from the Forest Gallery. Groups ‘c’ and ‘d’ overlap and contain all of the other collections. There was a positive correlation between the proportion of *Aaf* among *Ae. aegypti s.l.* and the proportion of mosquitoes with a midgut escape barrier for the 11 sites (Spearman's rank correlation; ρ_s_ = 0.797, P = 0.003).

### SNP discovery

Using the primers in Table 2, the regions of the Aminopeptidase N (Apn) (3.4.11.2) AAEL012783, α-amylase 2 (Amy2) (3.2.1.1) AAEL013421, α-Glycerophosphate dehydrogenase (aGPDH) (1.1.1.8) AAEL003873, Glucose-6-phosphate isomerase (GPI) (5.3.1.9) AAEL012994, Glutamate dehydrogenase (GluDH) (1.4.1.2) AAEL010464, Fumarase (Fum) (4.2.1.2) AAEL008167, and Phosphoglucomutase (Pgm) (5.4.2.2) AAEL010037 genes shown in Figure 3 were amplified in the 57 mosquitoes listed in Table 3. These were then screened for sequence variation using SSCP. All of the primers and the associated analyses for the *Early Trypsin* gene are published [45].

Figure 3 shows the region that was amplified with the PCR primers underlined. All SNP sites are also underlined and the chosen SNP site is in a box. Our selection of SNPs was biased in many ways. We only used SNP loci that demonstrated two alternate nucleotides because more nucleotides would require additional, more expensive SNP detection. In addition only those SNPs were used in which the most common allele had a frequency ≤0.95 among the 57 initial mosquitoes. The remaining SNPs were then screened as candidates for allele specific PCR. Each SNP was analyzed using Primer Premier 5.0® (Premier Biosoft International, Palo Alto, CA) to identify primers that would amplify a product ≤70 bp because this was the maximum size for discrimination by melting curve PCR. Furthermore, primers were eliminated that had potential to form hairpins or might anneal to one another.

αGPDH.55 is a synonymous G↔A transition in the third position of a Arg codon. Apn.1938 is a synonymous G↔A transition in the third position of a Gln codon. Amy2.447 is a synonymous G↔T transversion in the third position of a Pro codon, while Amy2.450 is a synonymous G↔T transversion in the third position of the adjacent Pro codon (Figure 3). Fum.-294 resides 294 bp upstream from the ATG start in the Fumarate hydratase gene. GPI. 1,500 is a synonymous G↔A transition in the third position of a Lys codon. GlutDH.507, 567, and 627 are all synonymous transitions in the third position of Val, Glu, and Iso codons, respectively. Pgm.954 is a synonymous A↔C transversion the third position of a Leu codon. TrypEarl detects a 13 bp deletion immediately 5′ to the ATG start in the Early Trypsin gene [45].

### SNP allele and genotype frequencies in collections

SNP allele frequencies were compared among and within years, subspecies, phytogeographic regions, vegetation zones and habitats by AMOVA [40]. We first tested whether alleles shifted in frequency among collection years (Table 5A) because this would have required partitioning by year any further analyses. Results indicate that 1% of the variation in allele frequencies arose among the three years and this was not significant in the permutation tests. All subsequent analyses, therefore, combined samples from different years.

**Table 5:** AMOVA of SNP allele frequencies among and within A) years, B) subspecies, C) regions, D) vegetational zones, E) phytogeographic regions, and F) habitats.

Source of variation	d.f.	Sum of squares	Variance Component	F	% variation
**A) Among collection years**					
Among years	2	16.6	0.0052	0.010	1.0
Among collections in years	16	91.3	0.0505	0.095 ***	9.4
Among mosquitoes in collections	972	428.3	−0.0408	−0.085	−7.6
Within mosquitoes	991	517.5	0.5222	0.028	97.2
Total	1981	1053.8	0.5371		
**B) Among subspecies in sympatry**					
Among six mixed collections	5	20.4	0.0437	0.080 ***	8.0
Between subspecies in collections	6	2.7	−0.0013	−0.003	−0.2
Among mosquitoes in collections	244	119.7	−0.0115	−0.023	−2.1
Within mosquitoes	256	131.5	0.5137	0.057	94.3
Total	511	274.4	0.5446		
Between subspecies	1	4.2	−0.0016	−0.087	−0.3
Among collections in subspecies	23	101.9	0.0531	0.100 ***	10.1
Among mosquitoes in collections	939	407.4	−0.0414	−0.003	−7.9
Within mosquitoes	964	498.0	0.5166	0.019	98.1
Total	1927	1011.5	0.5268		
**C) Among Northern, Central and Eastern Regions**					
Between regions	2	20.7	0.0072	0.013	1.3
Among collections in zones	16	87.2	0.0484	0.091 ***	9.0
Among mosquitoes in collections	972	428.3	−0.0408	−0.085	−7.6
Within mosquitoes	991	517.5	0.5222	0.028	97.2
Total	1981	1053.8	0.5371		
**D) Among three vegetational zones**					
Among three vegetational zones	2	15.9	0.0030	0.006	0.6
Among collections in zones	16	92.0	0.0510	0.096 ***	9.6
Among mosquitoes in collections	972	428.3	−0.0410	−0.085	−7.6
Within mosquitoes	991	517.5	0.5220	0.026	97.4
Total	1981	1053.8	0.5360		
**E) Among five phytogeographic regions**					
Among five phytogeographic regions	4	42.8	0.0173	0.032 *	3.2
Among collections in regions	14	65.1	0.0409	0.078 ***	7.6
Among mosquitoes in collections	972	428.3	−0.0408	−0.085	−7.6
Within mosquitoes	991	517.5	0.5222	0.032	96.8
Total	1981	1053.8	0.5397		
**F) Among four habitats**					
Among four habitats	3	24.8	0.0050	0.009	0.9
Among collections in habitats	15	83.1	0.0499	0.094 ***	9.3
Among mosquitoes in collections	972	428.3	−0.0408	−0.085	−7.6
Within mosquitoes	991	517.5	0.5222	0.026	97.4
Total	1981	1053.8	0.5364		

Next, we tested for variation in allele frequencies between the subspecies. In the first AMOVA we analyzed only the six collections in which the two subspecies were sympatric to avoid confounding differences among sites with differences among subspecies. Table 5B indicates that no variation was found between the subspecies. We then compared all *Aaa* collections with all *Aaf* collections, and again no variation was found between the subspecies. All subsequent analyses combined the subspecies in the six sympatric collection sites.

We next analyzed for variation among northern, central and eastern collections and Table 5C indicates that 1.3% of the variation in allele frequencies arose among the three regions but this was not significant in the permutation tests. All collections were next grouped into one of the three vegetation zones in Figure 2. Table 5D indicates that 0.6% of the variation in allele frequencies arose among these zones and that this was not significant. All collections were next grouped into the five phytogeographic regions (Table 1). Table 5E shows that 3.2% of the variation in allele frequencies arose among these regions and this was significant. Finally, all collections were grouped into the three habitat types (Table 1), and Table 5F indicates that 0.9% of the variation in allele frequencies arose among habitats and that this was not significant.

Table 6 lists Wright's F-statistics estimated using Weir and Cockerham's methods [44] for the entire study. F_ST_ estimates at each locus were significantly (P≤0.0001) greater than 0. The largest amount of variance was detected at the GlutDH.507 locus, the least occurred at the TrypEarl locus. Many F_IS_ estimates at each locus were significantly (P≤0.0001) greater or less than 0. Of 185 independent tests 56 were significant; far in excess of the nine expected with 5% Type 1 error rate. However, there was no general trend towards excess homozygotes (F_IS_>0) or excess heterozygotes (F_IS_<0). In half of the tests F_IS_>0 and in the other half F_IS_<0. The largest deviance in F_IS_ was seen at GlutDH.627 (F_IS_ = −0.276) with excess heterozygotes in six collections. The smallest deviance in F_IS_ was seen at GlutDH.507 (F_IS_ = −0.012) with a slight excess of heterozygotes in one collection.

**Table 6:** Wright's F-statistics estimated by Weir and Cockerham's method [44] among the 19 Senegal collections.

Locus	F_IS_ (F_IS_≠0/no.tests: F_IS_>0, F_IS_<0)	F_ST_	F_IT_
aGPDH.55	−0.023 (3/15: 2+, 1−)	0.100***	0.079
Apn.1,938	0.098 (6/19: 5+, 1−)	0.110***	0.197
Amy2.447	0.096 (6/18: 5+, 1−)	0.086***	0.174
Amy2.450	−0.166 (6/19: 1+, 5−)	0.116***	−0.031
Fum.-294	−0.050 (7/17: 4+, 3−)	0.146***	0.104
GPI.1,500	−0.143 (3/15: 2+,1−)	0.090***	−0.041
GlutDH.507	−0.012 (5/12: 4+, 1−)	0.209***	0.200
GlutDH.567	−0.183 (6/19: 1+, 5−)	0.090***	−0.076
GlutDH.627	−0.276 (7/18: 1+, 6−)	0.081***	−0.173
Pgm.954	−0.166 (6/18: 2+, 4−)	0.135***	−0.009
TrypEarl	−0.026 (1/15: 1+, 0−)	0.038***	0.013
Mean	−0.083 (56/185: 28+, 28−)	0.110***	0.035
JackKnife Mean	−0.084	0.109	0.035
Std. Dev.	0.047	0.01	0.045

***: P≤0.0001.

Under F_IS_ are indicated the number of tests for goodness-of-fit to Hardy-Weinberg expectation in which F_IS_≠0 over the number of tests. This is followed by the number of tests in which F_IS_>0 and the number in which F_IS_<0.

**Figure 6:**
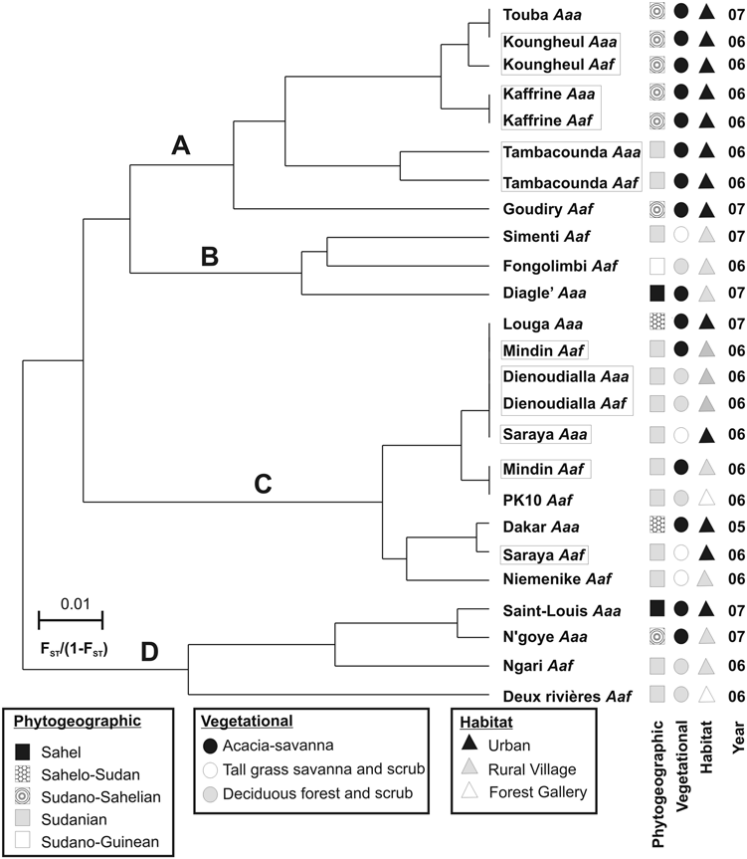
UPGMA cluster analysis of pairwise F_ST_/(1−F_ST_) markers among the 25 collections.

Unweighted pair-group method with arithmetic mean (UPGMA) cluster analysis [46] of pairwise F_ST_/(1−F_ST_) among the Senegalese collections (Figure 6) indicates four clusters labeled A–D. The collection year was distributed independently among clades (Fisher's Exact Test (FET), p = 0.1397). Subspecies were distributed independently among clades (FET, p = 1.0000). The vegetative zone in which the collection was made was also independent among clades (FET, p = 0.0643). However, collections were clustered by phytogeographic region (FET, p = 0.0010) and habitats (FET, p = 0.0068) with a disproportionately large number of Urban and *Acacia* Savanna collections occurring in Clade A. Thus, aside from habitats, the cluster analysis largely confirms the AMOVA results.

**Figure 7:**
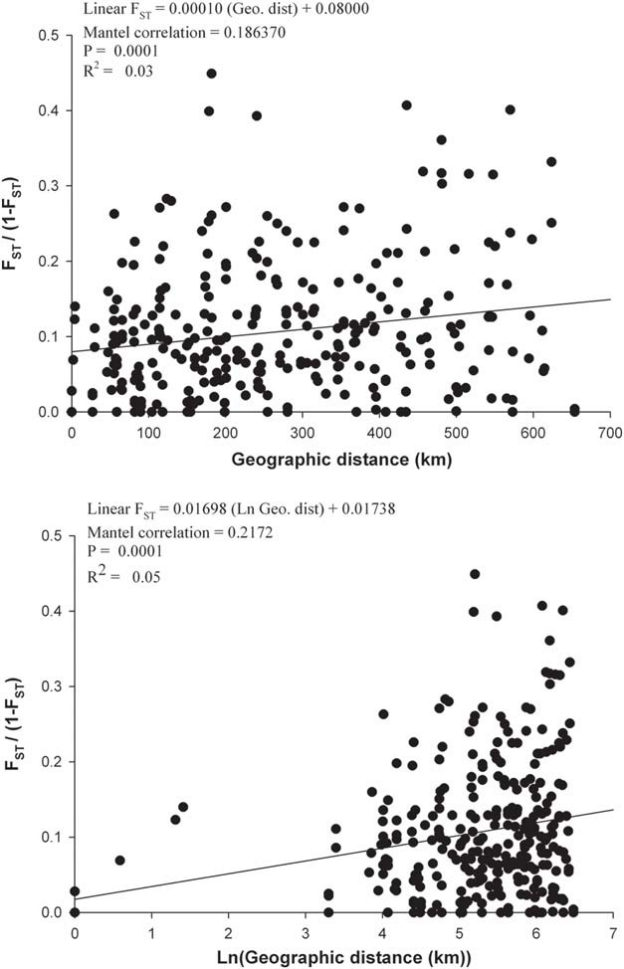
Regression analysis of pairwise F_ST_/(1−F_ST_) for the SNP markers against geographic distances (km) (upper panel), pairwise F_ST_/(1−F_ST_) for SNP markers against ln(geographic distances (km)) (lower panel).

A Mantel analysis of pairwise F_ST_/(1−F_ST_) against geographic distances indicated a highly significant correlation between genetic and geographic distances among collections (Figure 7). While a significant correlation is usually interpreted as evidence of isolation by distance, the regression coefficients were small (R^2^ = 0.03–0.05) and general inspection of the data points in the untransformed geographic distance graph suggests only a weak trend.

## Discussion

We have demonstrated a northwest–southeast cline in the abundance of *Aaa* and *Aaf* in Senegal as determined by the number of pale scales on the first abdominal tergite of individual mosquitoes. The vector competence of mosquitoes in some of these collections was analyzed for susceptibility to DENV-2 susceptibility and was correlated with the distribution of the two subspecies. Population genetic analyses with SNPs revealed large and significant differences in allele frequencies among collections. However, none of this variation was attributable to the year of collection, subspecies, the vegetation zone, or the habitat in which the collections were made. Minor amounts of the variation in allele frequencies were attributable to the geographic distance among collection sites and to the phytogeographic region in which the collections were made.

Huber *et al.* [47] recently published an in-depth examination of gene flow among five cities in Senegal using variation at 10 isozyme markers. They collected five samples from Barkedji in the Sahel; Diourbel, Kaffrine and Koungheul from the Savannah region; and Kedougou from the Forest gallery for a total of 25 samples containing 1,086 mosquitoes. Their overall F_ST_ value was 0.078. Most (74%) of F_ST_ was accounted for by variation among the five collections within each city, while the remainder was accounted for by differences among the five cities. Our overall F_ST_ value was slightly larger (0.109) but we did not compare multiple collections within cities; some of our sites had small sample sizes (which inflate F_ST_ estimates [48]) and our study included 19 sites over a much larger geographic range. Huber *et al.* [47] also performed an AMOVA among collections in the same vegetation zones as in Figure 2 and, as with our study, more variation arose within (5.5%) rather than among (2.6%) zones. Huber *et al.* also performed an AMOVA on subspecies. As with our study, more of the variation arose among collections within a subspecies (5.7%) rather than among subspecies (3.6%). However, even though this was a small percentage, it was significant in their permutation tests. We only examined gene flow in the six collections where the subspecies are sympatric and found a non-significant 1.4% of frequency variation arose between subspecies. In contrast Huber *et al.* compared Kedougou (*Aaf*) with all other cities (*Aaa*). Thus their subspecies variance included, and was therefore inflated by, variation among cities. Huber *et al.* performed a cluster analysis of linear F_ST_ values and, as in Figure 6, found no clusters corresponding to cities, subspecies or vegetation zones. They also tested for isolation by distance using the same analyses as presented here and found none. While our regression was significant, the linear regression model explained little of the overall variance.

There is a major discrepancy between our F_IS_ results and those of Huber *et al.* The number of significant tests in their study was the number expected with a 5% Type 1 error rate but the number of significant tests in our study was far in excess of this expected rate. This initially suggested to us that our melting curve PCR assay was inaccurate. The assay might not be equally sensitive to both nucleotides at a locus and thus indicate an apparent homozygote for one allele in mosquitoes that are in reality heterozygotes, thus yielding F_IS_>0. The assay might also not be specific and thus indicate an apparent heterozygote in mosquitoes that are in reality homozygotes, thus yielding F_IS_<0. The problem with this interpretation is that F_IS_ = 0 for the majority of tests at each locus and F_IS_ was not consistently greater or less than zero in any one collection or at any one locus. Nevertheless, we amplified and sequenced PCR products from 2–3 individuals in a collection and at a locus where F_IS_≠0 and in every case confirmed the genotype reported by melting curve PCR assay. In addition, we reviewed our initial sequence results from some of the 57 mosquitoes listed in Table 3. These also did not conform to Hardy-Weinberg expectations. Sometimes there was an excess of homozygotes at a locus but for other loci there was an excess of heterozygotes. At this time, we have no explanation for this discrepancy.

Both studies agree that very little or no variation exists between the subspecies. This is in stark contrast to similar studies [25] done in East Africa where allozyme frequencies differed markedly between the subspecies. Our results were presaged by McClelland [10] who found intermediate forms in areas of sympatry. These forms exhibited a wide range of pale scaling and occurred in peridomestic habitats. More recently, mosquitoes morphologically consistent with *Aaf* were found breeding domestically indoors in Nigeria [12] and Gabon [13]. Huber *et al.* [47] readily identified both forms in Senegal. Therefore, the classic behavioral/habitat descriptions given by Mattingly [8] for these two subspecies are not valid throughout Africa.

**Figure 8:**
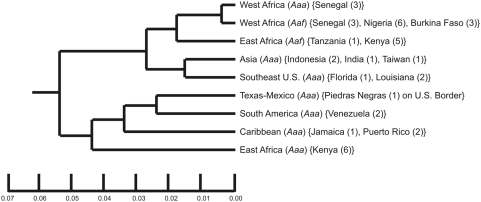
Addition of Senegal collections to Figure 1.

This tautology between *Aaa* and *Aaf* in West Africa therefore suggests a revision to Figure 1 in which West African *Aaa* and *Aaf* are monophyletic within the upper clade (Figure 8). This revision suggests three fundamental conclusions. First, because *Aaf* is only found in Sub-Saharan Africa, and West African *Aaa* and *Aaf* are monophyletic, our results strongly support Mattingly's original suggestion [9] that *Aaa* arose from a sylvan *Aaf* population probably in West African forests. Second, Asian and Southeastern US *Aaa* populations originated from West Africa *Aaa* rather than *Aaf* as was previously suggested [27]. Third, West African *Aaa* subsequently spread into East Africa where they adapted to human habitats, and subsequently gave rise to the Texas/Northeastern Mexico, Caribbean, and South American *Aaa*.

In agreement with the early literature [17]–[19], we also found that *Aaf* had significantly lower vector competence than *Aaa*. Among pure *Aaa* collections, the disseminated infection rate (DIR) was 73.9% with a midgut infection barrier (MIB) rate of 6.8%, and a midgut escape barrier (MEB) rate of 19.3% while among pure *Aaf* collections, DIR = 34.2%, MIB rate = 7.4%, and MEB rate = 58.4%. These patterns are consistent with those reported earlier for the two subspecies with YFV and DENV1-4 [17]–[19],[49], but are inconsistent for specific locations. DENV-2 virus has been isolated from both western Senegal (*Bandia Village in Figure 2) [50] and extensively from the Kédougou area in eastern Senegal (near Ngari in Figure 2) [51],[52]. However, a comprehensive serosurvey for DENV exposure has not been made and so we cannot test for a correlation between *Aaa* abundance and risk for DENV exposure.

When Tabachnick *et al.* [17] examined the susceptibility of “West African Sylvan” populations from Dakar and N'goye to YFV infection they found the DIR to be 11 and 7% respectively. This is odd in two respects. First we found no *Aaf* in our Dakar and N'goye collections, and secondly, the DIRs with DENV-2 were 50 and 90% respectively. It is possible that vector competence for the long passaged Asibi strain of YFV used by Tabachnick *et al.* [17] is low (their most competent population only had a 53% DIR). But it is also possible that the subspecies composition of these sites has changed.

A group at Institut Pasteur de Dakar published a paper in 2008 [53] also measuring vector competence of *Ae. aegypti s.l.* populations from six locations in different bioclimatic zones and habitats of Senegal. They examined competence using a sylvatic (ArD 140875) and an epidemic (ArA 6894) DENV-2 isolate. They found that Senegalese *Ae. aegypti s.l.* populations had a high MIB rate (74–100%) and a highly variable DIR (10–100%). Both their study and ours examined vector competence in Dakar and N'goye and their findings are completely incongruent with ours. We believe three factors explain the discrepancies. First, they did not use standard susceptible and refractory strains as controls. Thus they have no baseline for comparison. Secondly, their MIB rates were very high resulting in DIR estimates based on ≤2–10 midgut-infected females. Third, their TCID50/ml titers were 10^6.5–7.0^ plaque forming units (pfu) while we used titers of 10^7.5–8.5^ pfu and Tabachnick *et al.* [17] used YFV TCID50/ml titers of 10^7.8–8.8^ pfu. Their low DIR was therefore probably due to low blood meal titers of both DENV-2 isolates.

Taken as a whole, our descriptions of subspecies distributions, vector competence and allele frequencies provide a very incomplete picture. In fact, they present a paradox. Why are the distributions of subspecies and vector competence rates distributed along a northwestern-southeastern cline while no such pattern is seen with either isozymes or SNPs? Why are SNP or isozyme phylogenies not distributed along the same cline? Our current knowledge of the distribution and vector competence of the two subspecies in West Africa in general and in Senegal in particular is still very incomplete. An additional deficiency in the current study is that no data were collected as to feeding, resting, or oviposition behaviors exhibited by mosquitoes at each sites. In addition, Figures 4 and 5 suggest a northwest-southeast cline in subspecies composition and vector competence but, in fact, the sampling locations were mostly distributed from northwest to southeast. Note that there are no collections from the northern or western marshes, the southern broadleaf evergreen forest, the western tall grass savanna and scrub, nor from the western deciduous forest and scrub south of The Gambia. A broader study of subspecies, vector competence and allele frequencies throughout West Africa may provide clues towards resolving this paradox.
